# Fusing pre-trained convolutional neural networks features for multi-differentiated subtypes of liver cancer on histopathological images

**DOI:** 10.1186/s12911-022-01798-6

**Published:** 2022-05-04

**Authors:** Xiaogang Dong, Min Li, Panyun Zhou, Xin Deng, Siyu Li, Xingyue Zhao, Yi Wu, Jiwei Qin, Wenjia Guo

**Affiliations:** 1 Department of Hepatopancreatobiliary Surgery, Cancer Affiliated Hospital of Xinjiang Medical University, Ürümqi, Xinjiang China; 2 Key Laboratory of Signal Detection and Processing, Xinjiang University, Ürümqi, 830046 China; 3 College of Information Science and Engineering, Xinjiang University, Ürümqi, 830046 China; 4 College of Software, Xinjiang University, Ürümqi, 830046 China; 5 Cancer Institute, Affiliated Cancer Hospital of Xinjiang Medical University, Ürümqi, 830011 China; 6 Key Laboratory of Oncology of Xinjiang Uyghur Autonomous Region, Ürümqi, 830011 China

**Keywords:** Liver cancer, Histopathological images, Feature fusion, FuNet

## Abstract

Liver cancer is a malignant tumor with high morbidity and mortality, which has a tremendous negative impact on human survival. However, it is a challenging task to recognize tens of thousands of histopathological images of liver cancer by naked eye, which poses numerous challenges to inexperienced clinicians. In addition, factors such as long time-consuming, tedious work and huge number of images impose a great burden on clinical diagnosis. Therefore, our study combines convolutional neural networks with histopathology images and adopts a feature fusion approach to help clinicians efficiently discriminate the differentiation types of primary hepatocellular carcinoma histopathology images, thus improving their diagnostic efficiency and relieving their work pressure. In this study, for the first time, 73 patients with different differentiation types of primary liver cancer tumors were classified. We performed an adequate classification evaluation of liver cancer differentiation types using four pre-trained deep convolutional neural networks and nine different machine learning (ML) classifiers on a dataset of liver cancer histopathology images with multiple differentiation types. And the test set accuracy, validation set accuracy, running time with different strategies, precision, recall and F1 value were used for adequate comparative evaluation. Proved by experimental results, fusion networks (FuNet) structure is a good choice, which covers both channel attention and spatial attention, and suppresses channel interference with less information. Meanwhile, it can clarify the importance of each spatial location by learning the weights of different locations in space, then apply it to the study of classification of multi-differentiated types of liver cancer. In addition, in most cases, the Stacking-based integrated learning classifier outperforms other ML classifiers in the classification task of multi-differentiation types of liver cancer with the FuNet fusion strategy after dimensionality reduction of the fused features by principle component analysis (PCA) features, and a satisfactory result of 72.46% is achieved in the test set, which has certain practicality.

## Introduction

Liver cancer has developed into one of the most common and fatal malignancies due to its high incidence and mortality rate, which endangers human health seriously [1]. According to the latest global cancer burden data, in 2020, it is estimated that there were 19.3 million new cancer cases and 10 million deaths from cancer worldwide, of which 830,000 were from liver cancer, making liver cancer the most common cause of cancer death after lung cancer [2]. According to the WHO classification system, the differentiation status of tumor cells between malignant and normal cells can be better determined by analyzing the differentiation type of tumor, so that the malignancy degree and growth cycle of patients can be better evaluated, and the best treatment plan for patients can be clarified. According to research, it is known that liver cancer patients are prone to different prognosis due to their different differentiation degrees, and liver cancer with Poorly differentiation tends to be more aggressive, and treatment plans differ greatly from those of well and moderate differentiated tumors, which have poor prognosis and often lead to a lower survival rate [1]. When facing tumor patients with different differentiation degrees, it is especially important to efficiently evaluate the differentiation degree of liver cancer patients so as to adopt timely and effective treatment plans. Therefore, this study has important clinical significance for exploring different types of differentiation of primary liver cancer.

Imaging modalities such as computed tomography, ultrasound, magnetic resonance imaging, and various preoperative laboratory tests can be an important reference in cancer detection for diagnosis and staging [3]. However, histopathological image analysis (HIA) is the gold standard for tumor qualitative and clinical diagnosis. HIA is a key step in achieving the goals of early detection, diagnosis, and treatment of liver cancer [4], which is usually performed by pathologists through visual observation, but this process is time-consuming, tedious, and easily limited by the experience of pathologists themselves. Therefore, it is more necessary to implement an automated HIA for liver cancer which can improve the accuracy and efficiency of diagnosis [5].

Currently, the availability of a large number of medical images has made it possible to automatically analyze computer-assisted liver cancer images and accelerate the diagnostic efficiency of pathologists [1]. This is a very challenging task when less experienced physicians analyze thousands of medical images, which are prone to misses and misdiagnosis. Therefore, it is very difficult to rely solely on physicians for visual analysis. However, computer-aided diagnostic methods have the advantages of saving time, speed, and objective results compared to physicians' visual discrimination methods. Inevitably, however, computer-aided methods still have many drawbacks: image information such as histological features used to express lesions is rich in meaning, which makes texture descriptors and statistical feature descriptors require autonomous setting by computer personnel with specialized knowledge if more comprehensive information is to be obtained from the images themselves [3, 6–8], and are susceptible to personal subjective judgment in feature extraction. Traditional machine learning models are bulky when facing with different types of data. They cannot be adaptive to learn features because of the inconsistency of important features among different types of datasets. In addition, there are many types of classifiers with their own unique classification characteristics, so the same dataset still shows different classification effects with different models.

With the rapid development of deep learning techniques, both adaptive feature learning and automated medical image analysis have been substantially improved [9–11]. Numerous researchers have used CNNs to automatically extract image features, which abandon the aforementioned traditional and tedious hand-designed feature extraction methods, and the trained models can almost efficiently identify experimental objects with sufficient amount of data, and even the objective diagnostic results obtained by some excellent researchers can reach a level comparable to the results of diagnostic experts [1]. However, the automated analysis of digital histopathology images is still a challenging task in the following aspects: First, the number of available public medical image datasets with complete markers is very small. Second, there are significant color differences and size variation between some images. Third, it’s also affected by some extraneous objective factors such as noise, the use of patch level and whether all tumor regions are in image levels [12]. In order to address the above issues with better extend the automated research work on HIA of liver cancer, numerous researchers have conducted targeted exploratory experiments. Wang et al. tried to combine the whole slide images (WSIs) and machine learning methods, and proposed a patch-based convolutional neural network based on 60 liver tumor WSIs to better predict normal or tumor categories. In addition, they designed four sets of experiments to obtain the best classification effect [13]. Sun et al. noticed the problems of histopathological image analysis (HIA) in the early diagnosis of liver cancer. In order to solve these problems, they proposed a method for liver cancer histopathological image classification using only global labels. This study solved the problems of insufficient training samples of liver cancer histopathological image and large-scale image processing. Using transfer learning and multi-instance learning methods, patch-level features and image-level features are obtained, which can effectively distinguish abnormal or normal liver cancer histopathological images, thus providing help for the early diagnosis of liver cancer [3]. Wang et al. proposed a one-dimensional convolutional neural network based on the hyperspectral data obtained on hepatocellular carcinoma (HCC) sample slices, and used a weighted loss function to better improve the performance of the model. This method achieved a good classification effect on their data set, in which the area under receiver operating characteristic curve, sensitivity and specificity all reached more than 85% [14].

With the advent of deep learning, the research of digital pathology is advancing. In the past research work, we have witnessed many new methods for feature extraction based on deep learning pre-trained convolutional neural networks. Their wide application in different research is also similar to the recent ones in liver cancer, cervical cancer, Alzheimer’s disease, new coronary pneumonia, prostate disease and breast cancer diagnostics, even involving the identification of flower species and the classification of underwater images. In addition, we summarized relevant research papers published in recent years, and the results are shown in Table 1.

**Table 1:** Literature review of computer aided diagnosis for feature extraction using pre-trained convolutional neural networks

Work title	Research task	Model	Advantages and disadvantages
[15] Efficient deep features selections and classification for flower species recognition	Flower species recognition	AlexNet and VGG16	The research explored the similarity and intra-class variability among flower classes. The automatic flower recognition and classification were effectively realized by the method of pre-training neural network for feature extraction. It showed good performance on the Flower17 data set and Flower102 data set. However, this study only used two pre-trained neural networks for comparative experiments, AlexNet and VGG16, and failed to fully explore the feature complementarity between different pre-trained neural networks, and which caused a problem of incomplete experimental models
[16] Transfer learning with pre-trained deep convolutional neural networks for the automatic assessment of liver steatosis in ultrasound images	Automatic assessment of liver steatosis in ultrasound images	Inception-v3 and VGG-16	The study constructed 629 liver image data sets of two types of normal and liver steatosis, which evaluated two pre-trained convolutional neural network models using fine-tuning methods and obtained satisfactory results. However, there is still the problem of incomplete experimental models
[17] Few-shot hypercolumn-based mitochondria segmentation in cardiac and outer hair cells in focused ion beam-scanning electron microscopy (FIB-SEM) data	Few-shot hypercolumn-based mitochondria segmentation	VGG-16	This research uses the convolution features of a pre-trained deep multi-layer convolutional neural network (such as VGG-16) to realize a few shots automatic segmentation method of mitochondria in electron microscope images. The proposed method proves that it can still provide competitive performance with less training data. However, this study only uses VGG-16 as the feature extraction model, which caused a problem that the experimental model is not comprehensive
[18] ResFeats: Residual network based features for underwater image classification	Underwater image classification	ResNet-50	The study explored how to use pre-trained deep networks to classify and transfer learning underwater images, and strongly verified that combining res expertise from different layers can generate a powerful image descriptor. However, the complementarity of features between different levels of other pre-trained neural network models can still be discussed later
[19] Can pre-trained convolutional neural networks be directly used as a feature extractor for video-based neonatal sleep and wake classification	Video-based neonatal sleep and wake classification	VGG16, VGG19, InceptionV3, GoogLeNet, ResNet and AlexNet	The research also uses pre-trained convolutional neural networks (CNNs) as feature extractors, and compares the different classification effects between multiple pre-trained models. However, this study uses AlexNet to use Fluke (RGB) video frames with accuracy, sensitivity, and specificity of 65.3%, 69.8%, and 61.0%, respectively. There is still a lot of room for improvement, and a special neural network is proposed to train newborn data
[20] CovH2SD: A COVID-19 detection approach based on Harris Hawks optimization and stacked deep learning	COVID-19 detection	ResNet50, ResNet101, VGG16, VGG19, Xception, MobileNetV1, MobileNetV2, DenseNet121 and DenseNet169	The research focused on the impact of new coronary pneumonia. It used deep learning and pre-training models to extract features from CT images. The experimental process was complete and had good results. However, we hope that subsequent research can be extended to other types of medical images for extensive experiments

In view of the insufficient available public image data resources and complex clinical features of liver cancer histopathology images, this study uses a collected histopathology image dataset of liver cancer with multiple differentiation types and adopts a classification method based on pre-trained model feature fusion for liver histopathology images with multiple differentiation types. Feature learning was carried out using all and some of the convolutional bases in the pre-trained model separately for comparison experiments, and the method was applied to its own histopathology images of liver cancer. Then the features were fused using serial fusion to form image-level features for classification to achieve an automatic analysis process. The advantages of the method are as follows: With the help of two experienced pathologists, our method identifies tumor regions with specific manifestations in tissue sections first, and then the PRECICE 500B digital pathology imager was used to scans the tumor regions at 40 times. A complete whole slide images (WSIs) can contain more than 100,000 × 100,000 pixels, which is very challenging to analyze such images directly [3]. Therefore, in this study, only tissue within the tumor region was selected six histopathology images were extracted for imaging using a non-overlapping approach, thus ensuring that our own histopathology image dataset of liver cancer was composed of separate images of the whole tumor region. (2) To ensure the adequacy of the study, according to the research, we used the more common data enhancement methods (translation, rotation, flip) to expand the training set of histopathology images tenfold in an attempt to attenuate the effects of overfitting phenomena and to better address the problem of insufficient training data in the histopathology image dataset of liver cancer. (3) We use pre-trained CNN models to extract image features from liver cancer data and employ feature fusion to explore the complementarity of feature learning between models in an attempt to find the best image feature fusion method. In addition, we also cross-sectionally compare the different generalization performance with six classifier models, and finally find the best combination approach with the most suitable performance parameters.

## Materials and methods

This section presents information specific to the data sets included in the study and the specific methods and evaluation Indicators used in the experiments.

### Data collection

**Fig. 1:**
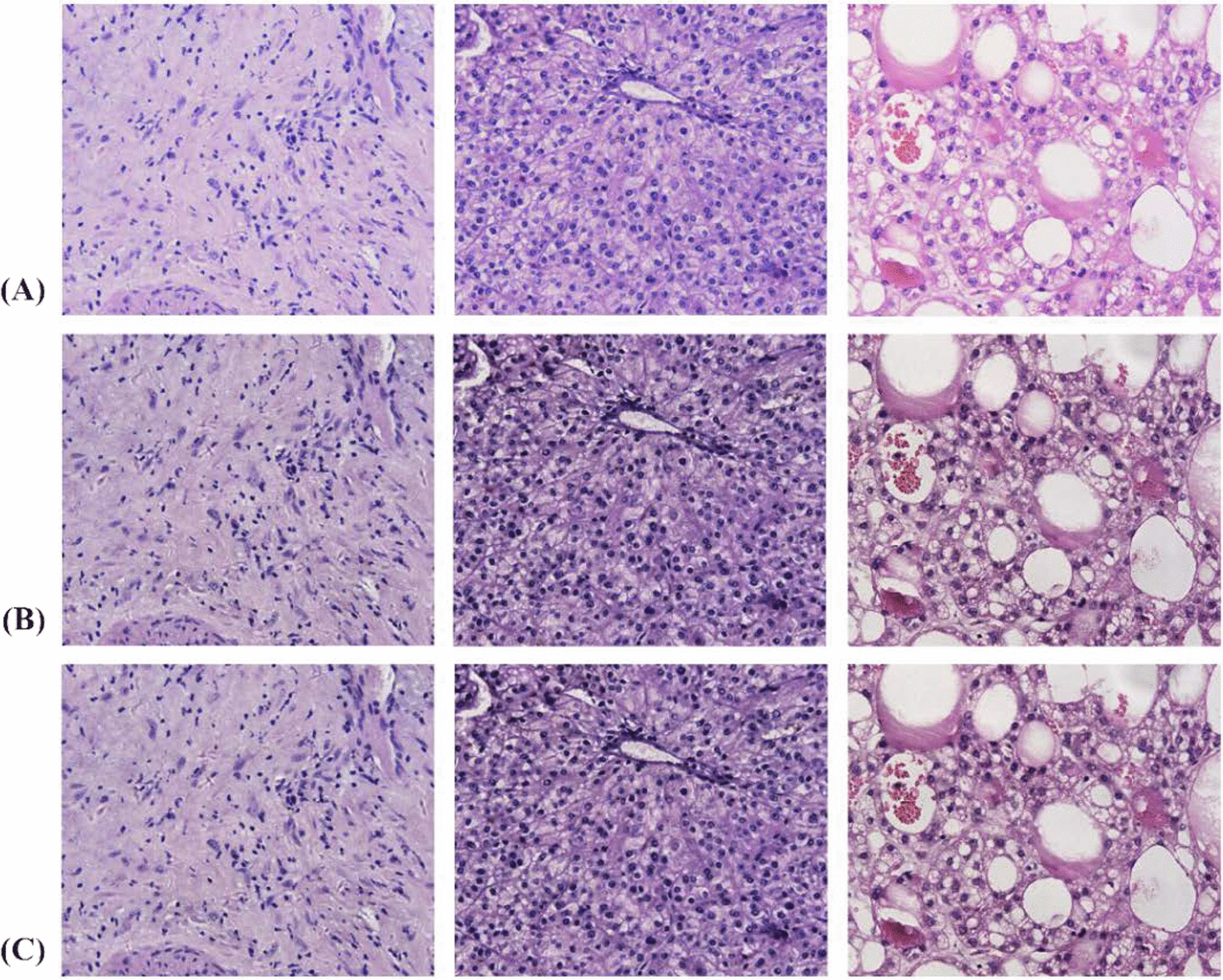
Randomly selected samples from the liver cancer datasets for demonstrate image processing. **a** Images of poorly differentiated, moderate differentiated and well differentiated tissues before the transformation. **b** Transformed images from (**a**) after AHE image processing. **c** Transformed images from (**b**) after Gaussian filtering operation

The dataset used in this study was provided by the Affiliated Cancer Hospital of Xinjiang Medical University to classify patients with primary liver cancer of three differentiation types: poorly differentiated, moderate differentiated and well differentiated. The relevant data included in this study were reviewed and approved by the Medical Ethics Committee of the Affiliated Cancer Hospital of Xinjiang Medical University and informed consent was obtained from the patients. All cases were liver tumor tissues diagnosed between 2010 and 2020. The dataset contained a total of 24 poorly differentiated, 27 moderate differentiated, and 22 well differentiated liver tumor histopathology image (all patients were confirmed by pathological findings and therefore included in the study). It is worth noting that in the context of medical imaging, marking the location of each lesion must be performed by experienced pathologists, which is a time-consuming and expensive process [21]. The histopathological images in this study were stained with hematoxylin and eosin staining (HE staining), and two experienced pathologists from our institution manually diagnosed the type of differentiation of tumor areas in patients with liver cancer and identified the tumor areas with specific manifestations in this tissue section. To enhance the generalization performance of the model, in this study, each sample was scanned at up to 40 times the power of the tumor region using a PRECICE 500B digital pathology imager according to the size of the region within the tumor [22], and finally only 6 histopathological images were extracted by selecting only the tissues within the tumor region in a non-overlapping manner, resulting in a total of 438 histopathological images collected. which were all stored at 1665 × 1393 pixels [23]. The alterations in the morphology of the nuclei with the three differentiation types are shown in Fig. 1, and the relevant information for all patients is presented in Table 2. The data set of this study was randomly divided in the ratio of 7:3 to generate the training set and the test set, thus ensuring the relative independence of the test set.

**Table 2:** Clinical profile of 73 liver cancer patients involved in the study

Characteristics	Complete dataset	Liver cancer differentiation		
		Poorly differentiated	Moderate differentiated	Well differentiated
No. of patient	73	24 (32.88%)	27 (36.99%)	22 (30.14%)
No. of images	438	144	162	132
Mean age		59.88	58.85	49.04
*Gender*				
Male	60 (83.33%)	17 (70.83%)	24 (88.89%)	19 (90.48%)
Female	12 (16.67%)	7 (29.17%)	3 (11.11%)	2 (9.52%)

Table entries are either the number of patients or the number of digital pathology images (percentage distribution in parentheses). There is one person in the well-differentiated type who has not yet clarified gender and age, so it is not shown in the table

### Image preprocessing

**Fig. 2:**
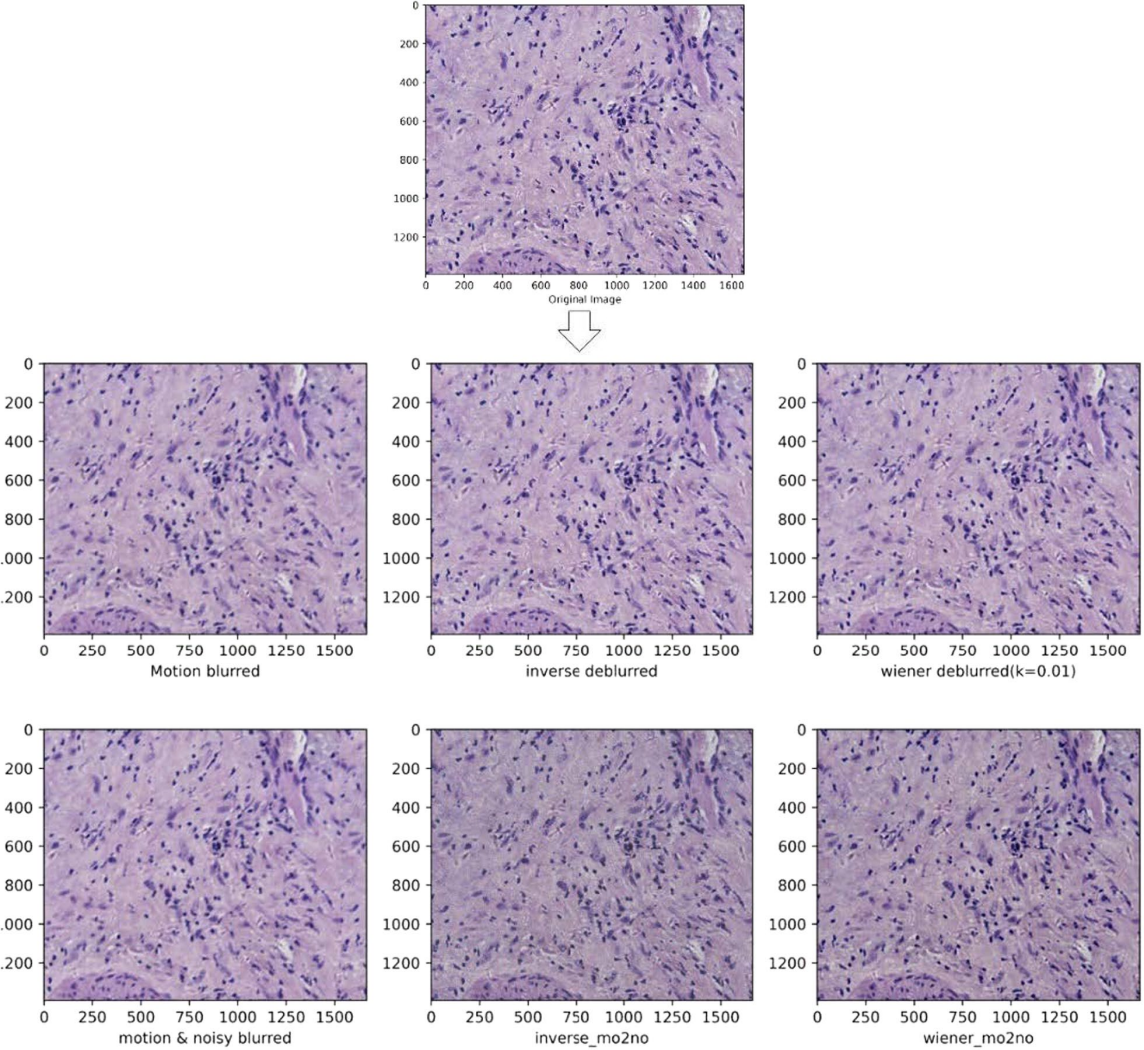
The images of poorly differentiated liver tumor tissue images after other processing methods are shown. From top to bottom and from left to right in the arrow refers to the content are: the pathologized images after simulated motion blur, and the detailed images after inverse filtering, wiener filtering, adding noise, adding noise after inverse filtering and wiener filtering are shown on their basis

**Fig. 3:**
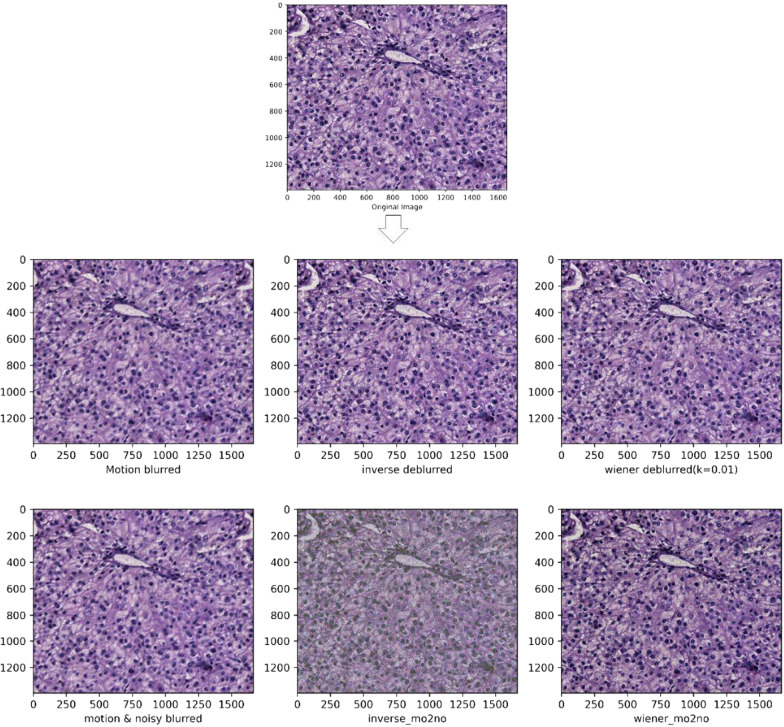
The images of Moderate differentiated liver tumor tissue images after other processing methods are shown. From top to bottom and from left to right in the arrow refers to the content are: the pathologized images after simulated motion blur, and the detailed images after inverse filtering, wiener filtering, adding noise, adding noise after inverse filtering and wiener filtering are shown on their basis

**Fig. 4:**
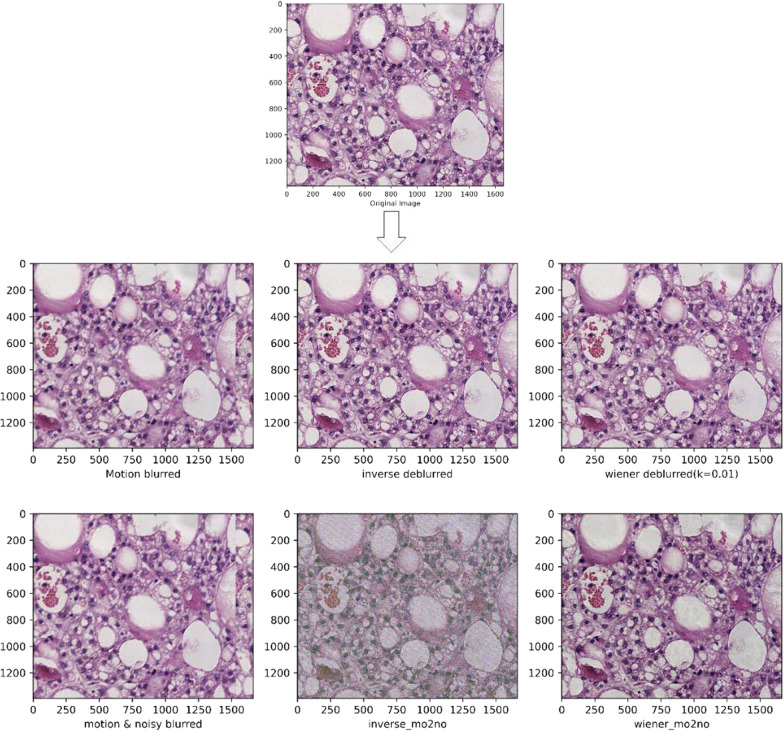
The images of well differentiated liver tumor tissue images after other processing methods are shown. From top to bottom and from left to right in the arrow refers to the content are: the pathologized images after simulated motion blur, and the detailed images after inverse filtering, wiener filtering, adding noise, adding noise after inverse filtering and wiener filtering are shown on their basis

It has been found that we are increasingly concerned about the negative impact of color differences between images, mainly in terms of limiting the accurate interpretation of tissue images by inexperienced pathologist, in addition, affecting the generalization performance improvement of the model. Therefore, in order to avoid the problem of image information loss due to the presence of excessive brightness in some regions of the image, this study used the adaptive histogram equalization algorithm(AHE) to effectively mitigate the problems such as color inconsistencies existing between images [3], which better adapts to the contrast of images due to unbalanced pixel value distribution by performing histogram equalization of responses to local regions, thus better preserves image details, improves the local contrast of the image and does not affect the overall image contrast. In addition, this study also explores various preprocessing methods to find a preprocessing method that better shows the image details. Firstly, Gaussian filtering is used to mitigate the negative effects of noise, as one of the efficient low-pass filters, mainly by replacing the pixel value at that point with the weighted average of the pixels in its neighborhood to better achieve noise reduction at the global scale [3, 12].Besides, we also compared the pathologized images after simulated motion blurring in a cross-sectional manner and showed separate images based on them with inverse filtering, Wiener filtering, images after adding noise, and inverse filtering with Wiener filtering after adding noise. The images after the two image pre-processing operations of adaptive histogram equalization and Gaussian filtering are shown in Fig. 1. The methods other than the above two data processing methods are shown in Figs. 2, 3 and 4. From Fig. 1, it can be seen that the images after the two preprocessing operations show smoother details and significant improvement in color differences, thus better preparing us for the subsequent study.

### Data augmentation

It has been found that analytical studies carried out on medical images are often subject to the occurrence of overfitting due to insufficient number of images [3]. Tables 3 and 4 summarize the review of data enhancement methods used in several different studies, from which it can be seen that several of the most commonly used strategies are rotation (4/18), flipping (4/18), cropping (4/18), cutting in the interior of the tissue block (6/18). According to the research, it was found that the performance was significantly improved when the models were trained using data enhancement, which verified the universality and wide applicability of data enhancement methods in the field of small samples of medical images from the side. To overcome this limitation, in this study, three different data enhancement strategies (rotation, translation and flip), which are more common, were used to generate liver tumor histopathology image datasets that could improve the size and quality of the training dataset and alleviate the overfitting problem [24]. The image enhancement rotation operations are done by rotating the images clockwise and counterclockwise by 45°, 60°, 90°, 210°, 240°, etc. Image flip is used to flip the image horizontally and vertically. Image translation is done by panning 10 pixels in each of the four direction: top, bottom, left and right [25]. Tables 5 and 6 show the dataset before and after data enhancement.

**Table 3:** Summary of data enhancement methods

Study	Method							
	Equalization	Cropping	Rotations	Flipping	Panning	Gray-scale processing	Random segmentation (select the image block)	Image segmentation (screening\ filtering)
All	1	4	4	4	1	2	6	2
[26]							X	
[27]								
[28]		X	X	X				
[29]								
[30]						X	X	
[31]						X		
[32]								
[33]							X	
[34]		X	X	X				
[35]	X							
[36]		X						X
[37]								X
[38]							X	
[39]							X	
[40]		X	X	X	X			
[41]								
[42]								
[43]			X	X			X

**Table 4:** Summary of data enhancement methods

Study	Method					
	Zooming	Color normalization (LAB\HSV\Other)	Color segmen-tation	Noise filtering processing	Image Normalization	Other
ALL	4	5	2	5	0	
[26]		LAB				
[27]	40X(boost)					
[28]	40X(boost)			Gaussian filtering Convolutional edge-enhanced filtering		
[29]		X				
[30]						
[31]		HSV		Denoise		
[32]				Denoise (Gaussian filtering)		
[33]					X	
[34]	X					
[35]	X	SVD				
[36]						
[37]				Denoise (Gaussian filtering)		
[38]				Denoise (Combining the median filter and the Gaussian filter)		
[39]						
[40]						
[41]			X			
[42]			X			
[43]		X

**Table 5:** Number of images without data augmentation

Images without data augmentation						
Class	Training set			Test set		
	Poorly differentiated	Moderate differentiated	Well differentiated	Poorly differentiated	Moderate differentiated	Well differentiated
Number	102	108	90	42	54	42

**Table 6:** Number of images with data augmentation

Images with data augmentation						
Class	Training set			Test set		
	Poorly differentiated	Moderate differentiated	Well differentiated	Poorly differentiated	Moderate differentiated	Well differentiated
Number	1020	1080	900	42	54	42

### CNNs

At present, with the structural improvement of CNNs, CNNs methods have been gradually applied to various tasks and fields, including image classification, target detection, face recognition, natural language processing and other related fields with remarkable effects. So far, modern CNNs network architectures consist of five main components: convolutional layer, pooling layer, activation function, discard rate(optional), and fully connected layer [44].

When dealing with small medical image datasets, this paper adopts an efficient and commonly used approach: pre-trained CNNs models [45]. In this paper, the ImageNet dataset with a sufficiently large and general data volume (1.4 million labeled images, 1000 different classification categories) is selected, and the model performs feature learning on the huge number of ImageNet datasets, preserving the general features extracted from the underlying convolutional layer. It is found that applying the model pre-trained on the ImageNet dataset to the medical image domain often achieve better visual performance as well. Therefore, four more classical CNN architectures: VGG16, ResNet50, DensNet201, and InceptionRensNetV2 were selected and applied to their own dataset of liver tumor histopathology images [45]. The pre-trained network is used either: (a) as a feature extractor, or (b) for fine-tuning [46]. In this form of learning, the pre-trained model extracts features for solving the target problem [46]. Feature extraction is the use of the representations learned in the previous network to extract interesting features from new samples. The features are then fed to a new classifier and trained from scratch.

### Introduction to image features

**Fig. 5:**
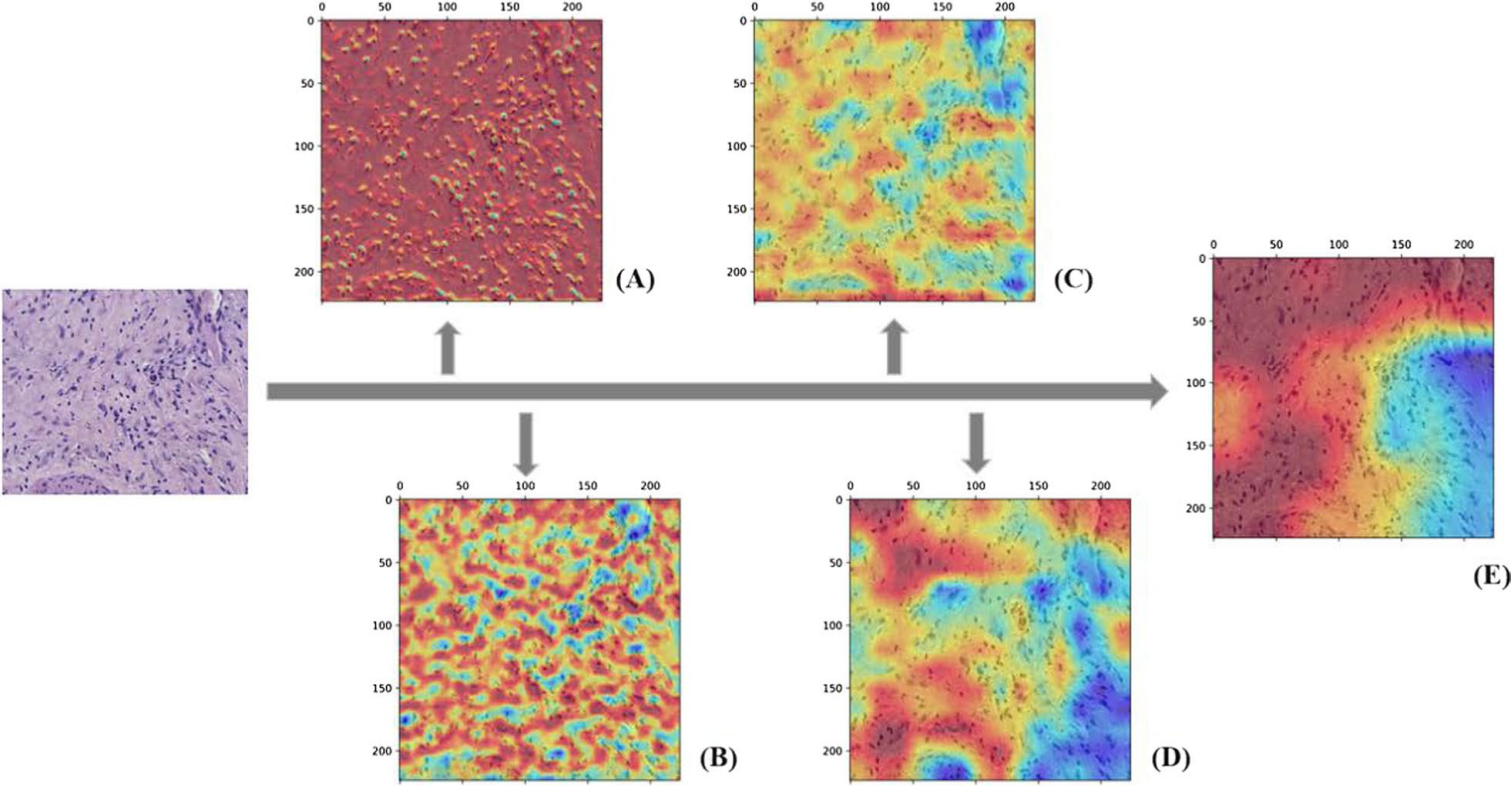
Feature vector displays each convolutional layer of ResNet50

This section elaborates on the process of extracting liver cancer features from the pre-trained neural network. Generally, different convolutional layers encode different aspects of the input image. The layers closer to the bottom of the neural network model can often extract more local and universal feature maps (such as corners, colors), the middle layer is easier to capture textures and shapes, and the layers closer to the top can extract more abstract and representative features [18]. In order to explain the differences in the features extracted between different levels in more detail, we take ResNet50 as an example to display the feature vectors of each level which is shown in Fig. 5. Due to the large differences between the dataset of multiple types of liver cancer and the original ImageNet dataset, this study attempts to find the similarities, degenerations and complementarities between features by exploring the fusion of feature vectors of different pre-training models and different levels [47]. We hypothesize that combining features from different training models and different levels can form a stronger and more representative image representation.

**Fig. 6:**
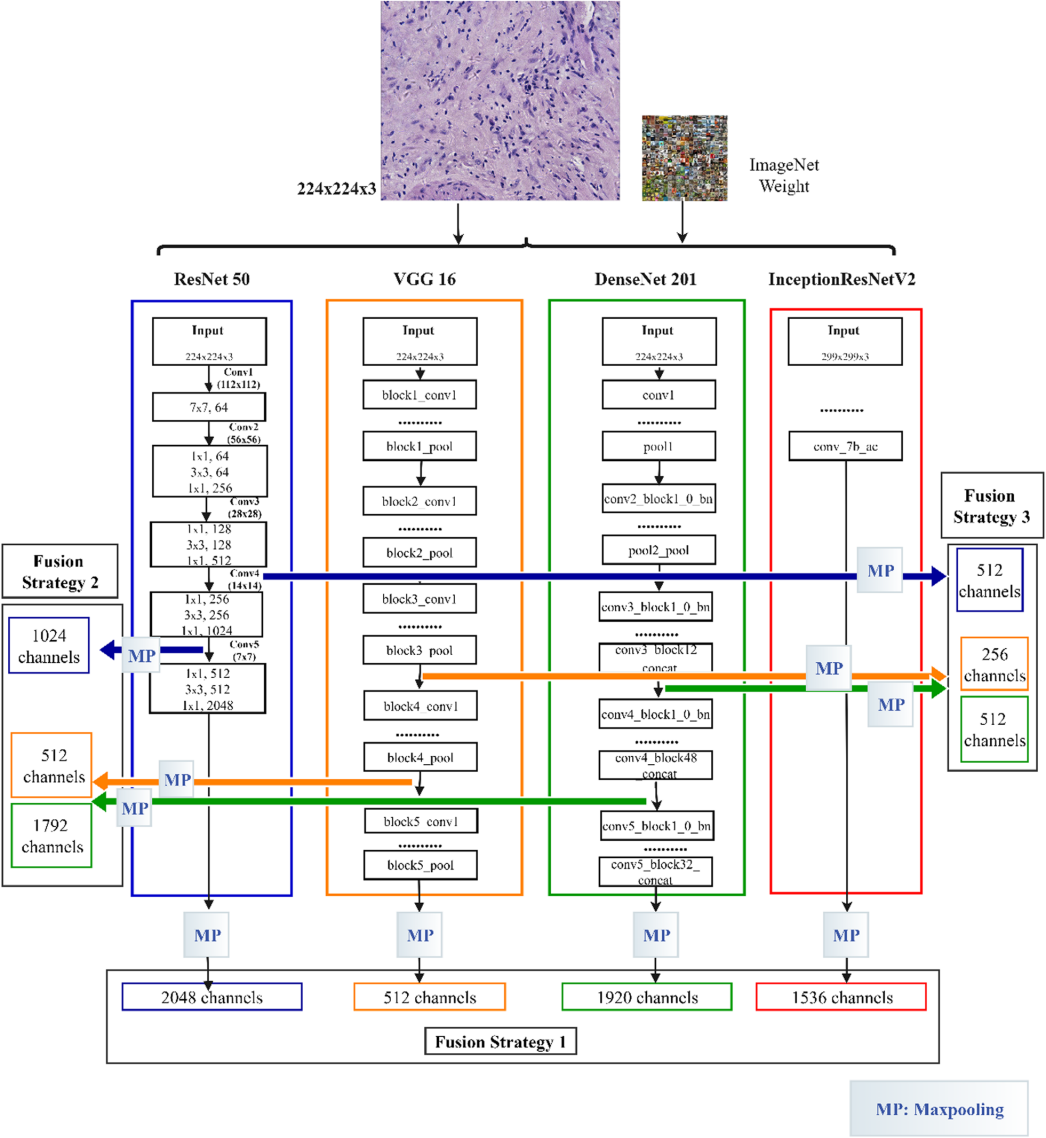
Extracting features from different layers in the different network and merge them

**Fig. 7:**
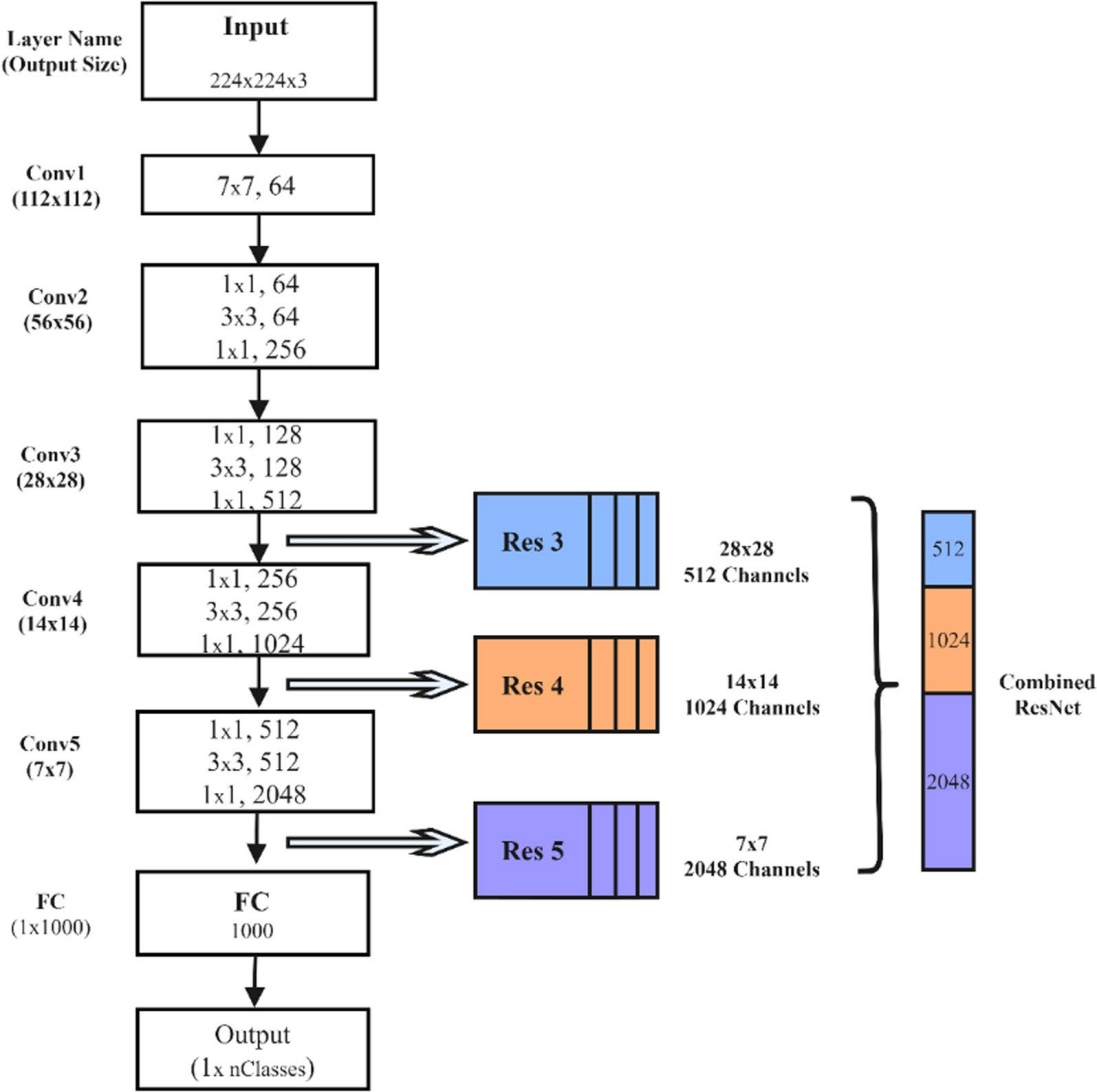
Extracting features from different layers in the ResNet network and merge them

**Fig. 8:**
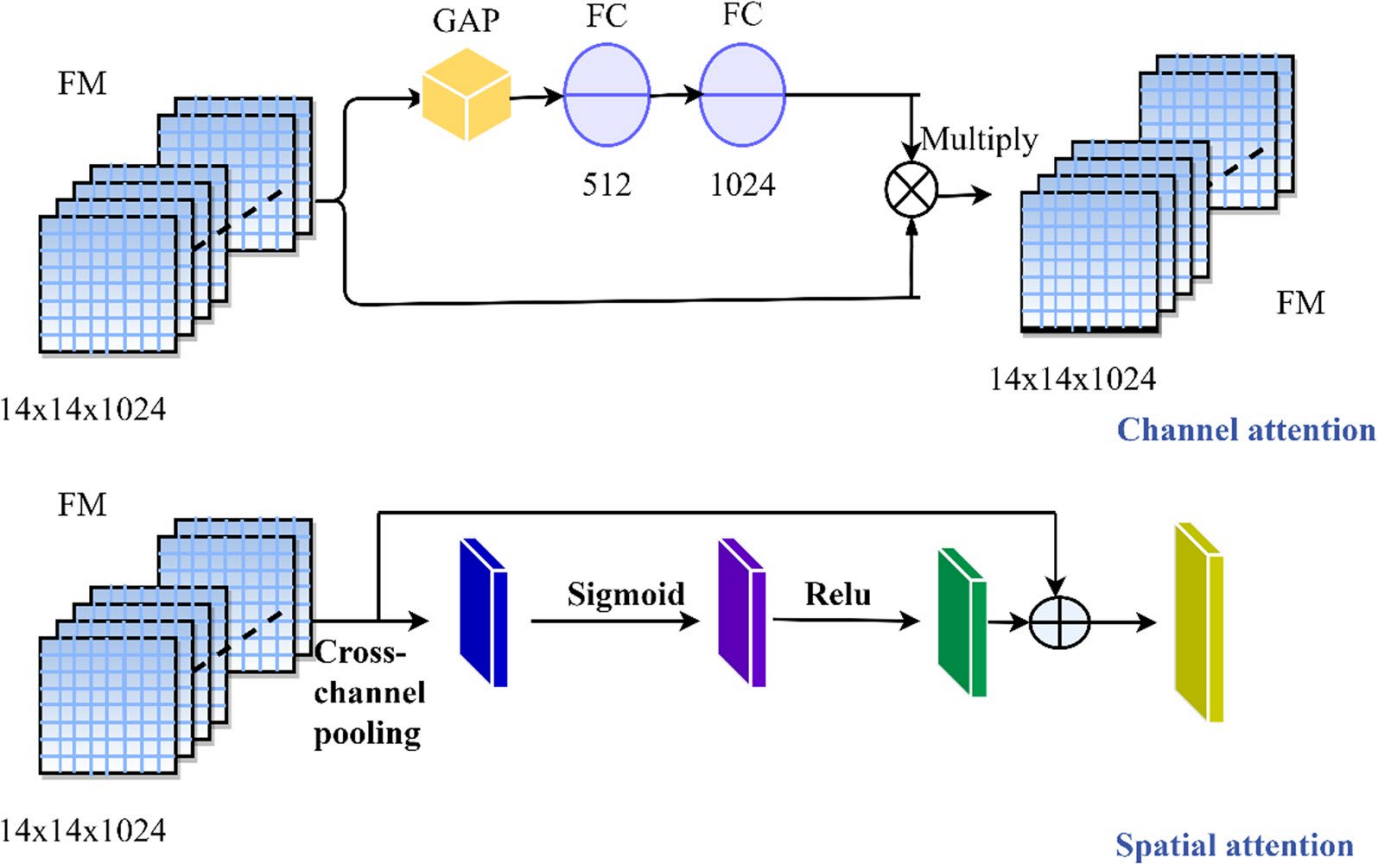
Components of the channel attention block and spatial attention block

**Fig. 9:**
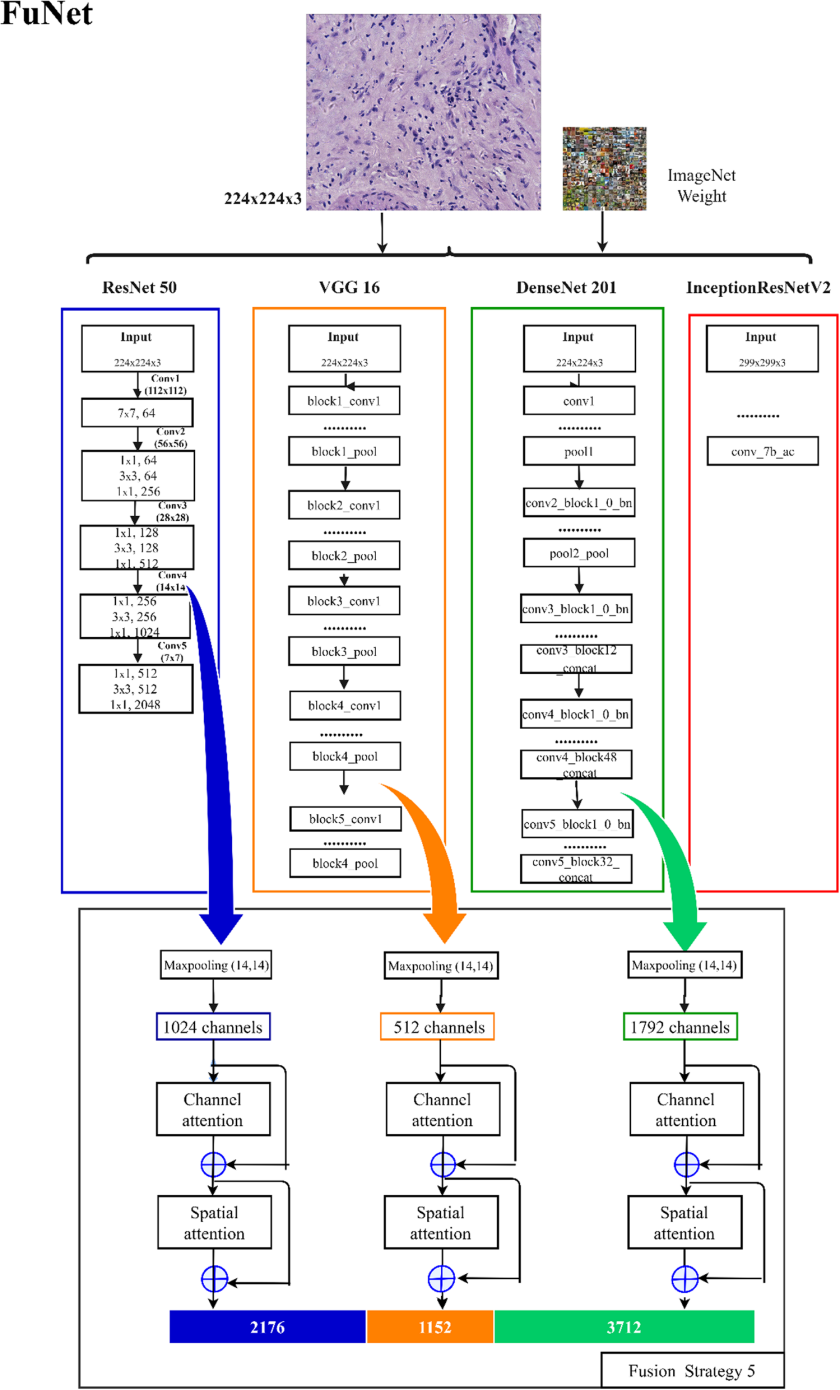
Extracting from the different layers of this network and the resulting combined FuNet are also shown

Thus we decided to do exploratory research with the following different fusion strategies: (1) On the basis of different pre-training models, we use all convolutional layers to realize feature extraction and fusion, as shown in Fig. 6, fusion strategy 1 [48]. (2) On the basis of the aforementioned strategy, we abandon InceptionRensENetV2 as the feature extractor, and use the partial convolutional layers of the other three pre-training models to extract and fuse features, as shown in Fig. 6, fusion strategy 2. (3) By using the feature output of the convolutional layer closer to the bottom, we explore the difference in classification results between the aforementioned three strategies, as shown in Fig. 6, the fusion strategy 3. (4) Considering that ResNet50 has 5 convolutional blocks, the output of the first two blocks does not encode any top-level abstract information, so we recommend fusing the output of the last three blocks to take advantage of mid-level and high-level features. We extracted the output of the last residual unit of the 3th, 4th and 5th blocks, and merged them as feature vectors after maximum pooling, then explored the fusion of the deep network based on the output feature vectors of the previous convolutional layer. Whether the latter convolutional layer can form a more representative feature vector representation is shown in Fig. 7 [18]. (5) In addition, we also pay attention to the attention mechanism that helps to capture most of the fine-grained features. The attention mechanism is beneficial to the research of computer vision tasks, and has been widely used in image classification, semantic segmentation, and so on. Therefore, this research proposes the FuNet model, which covers channel attention and spatial attention, and better suppresses channel interference with less information [49]. At the same time, the importance of each spatial position is clarified by learning the weight of different positions in the space [50]. It can be better applied to the classification research of the multi-differentiated types of liver cancer. The composition diagram of Channel attention block and Spatial attention block is shown in Fig. 8, and the structure diagram of FuNet is shown in Fig. 9. The classification results of the above experimental strategies will be reported in Sect. 3. It is worth noting that due to the limitation of external conditions such as memory, we added a maximum pooling layer of different specifications to the feature output layer for down-sampling. The number of channels of the feature vectors of different levels with different pre-training models finally extracted is shown in Table 7 shows. The number of box girder channels with different fusion strategies is shown in Table 8.

**Table 7:** Using different levels of convolutional bases as the level name and feature size of the feature extractor

Numbering	Combined content	Feature length
A1	ResNet50(full convolutional basis)	2048
A2	DenseNet201(full convolutional basis)	1920
A3	VGG16(full convolutional basis)	512
A4	InceptionRenseNetV2(full convolutional basis)	1536
B1	ResNet50(conv4_block6_out)	1024
B2	DenseNet201 (conv4_block6_out)	1792
B3	VGG16 (block4_pool)	512
C1	ResNet50(conv3_block4_out)	512
C2	DenseNet201 (conv3_block12_concat)	512
C3	VGG16 (block3_pool)	256

**Table 8:** The final feature vector size with different fusion strategies

Fusion strategy	Combined content	Feature length
Fusion strategy1	A1 ∪ A2 ∪ A3 ∪ A4	6016
Fusion strategy2	B1 ∪ B2 ∪ B3	3328
Fusion strategy3	C1 ∪ C2 ∪ C3	1280
Fusion strategy4	Res 3 ∪ Res 4 ∪ Res 5	3584
FuNet	B1 ∪ B2 ∪ B3 (Contain attention block)	7040

(∪ Denotes the serial fusion feature)

### Classifier

Support Vector Machine(SVM) is an algorithm developed gradually with the support of statistical theory, which has been widely used to solve problems in major fields, and has now developed into one of the mainstream machine learning algorithms [51]. SVM strives to achieve the lowest training error and testing due to its availability in the choice of classification models and model parameters [52]. The main goal of SVM is to find the "maximum interval" division hyperplane suitable for classifying samples [53], which makes the model generalize more and classify better. When dealing with small sample problems, SVM often shows unique classification performance compared to artificial neural networks. Besides, the existence of the kernel function implicitly defines the existence of a feature space in which we expect the samples to be linearly differentiable, so the choice of the kernel function becomes one of the variables affecting the classification performance of the support vector machine classifier. If an inappropriate kernel function is chosen, the samples are often mapped to an inappropriate feature space resulting in poor classifier performance. Therefore, this study investigates the different classification performance of the SVM classifier with the action of four kernel functions.

We also horizontally compared the classification performance between the KNN classifier and the random forest classifier. In addition, we use ensemble techniques (stacking, boosting and gradient boosting) to combine the classifiers together [54], which improves the classification accuracy by merging the classifiers in sequence [55, 56].

### Evaluation criteria

To better evaluate the reliability and generalization ability of models, in this paper, we use the receiver operating characteristic (ROC) curve to judge the performance of our built classification models and the area under ROC curve (AUC) to verify the generalization ability of the models in a more intuitive way [12, 48].

We combine the samples according to their true categories and the predicted categories of the learner, and the confusion matrix is composed of four main aspects: true positive (TP), false positive (FP), true negative (TN), and false negative (FN). Finally, the horizontal and vertical axes of the ROC curve are calculated, where the horizontal axis is the "false positive rate"(FPR) and the vertical axis is the "true case rate"(TPR), which are defined as Eqs. (1) and (2), respectively.

1 $${\rm FPR}=\frac{{\rm FP}}{{\rm TN}+{\rm FP}}$$

2 $${\rm TPR}=\frac{{\rm TP}}{{\rm TP}+{\rm FN}}$$

Besides, Precision, Recall, F1 value, etc. are various different Indicators to measure the performance of the classifier according to different calculation methods, and the formulae are shown in Table 9 [56].

**Table 9:** Model evaluation indicators

Evaluation index	Calculation formula
Accuracy	$\frac{{TP + TN}}{{TP + TN + FP + FN}}$
Recall	$\frac{{TP}}{{TP + FN}}$
Precision	$\frac{{TP}}{{TP + FP}}$
F1	$$\frac{{2*Precision*Recall}}{{Precision + Recall}}$$

## Results and discussion

### Experimental conditions

All relevant code for this study was developed in the python language, and we implemented the above steps using Keras under Tensorflow 2.0.0, a popular Python framework for deep learning [46]. The size of the original patch was 1665 × 1393 pixels and the patch was resized to 224 × 224 pixels to match the number of inputs of each neural network input layer [57]. All our subsequent experiments are to divide the expanded training set into a training set and a validation set to train the model, and then use an independent test set to test the classification performance of the model. The ensemble techniques (stacking, boosting and gradient boosting) used in this research are all from scikit-learn learning pants.

### Performance analysis: CNN feature analysis with full convolutional base

As can be seen from Table 10, the classification effect of a single feature extracted from a single model is poor, and the accuracy of the test set is around 50% mostly. Besides, when two single features are fused, the test set effect does not improve significantly and somehow even decreases. It indicates that when two single image features are fused, they may not complement each other well, which would cause feature redundancy and inapparent accuracy improvement. However, we can see that compared with the single feature extraction method of A1, A2, and A3, the validation set accuracy of the fusion model of A1 ∪ A2 ∪ A3 achieves 90% except for the Linear-SVM model and Sigmoid-SVM model, which is a satisfactory result. Meanwhile, the test set result achieves 63.04% with the fusion model of A1 ∪ A2 ∪ A3, which proves from the side that there is singularity in the extracted features when using a single pre-trained neural network model with a full convolutional basis as the feature extractor. And when fusing the feature vectors with multiple well-performing pre-trained neural networks, it improves the feature representation capability and effectively prevents having risk of poor performance of the single features extracted from a single model.

**Table 10:** Using full convolutional basis with different networks for feature extraction and fusion classification performance

Strategie	Classifer			
	A1	A2	A3	A4
Sigmoid-SVM	44.20/63.33	62.32/73.17	54.33/73.00	39.13/36.00
Rbf-SVM	62.32/98.67	58.70/91.83	55.80/87.00	39.86/36.33
Poly-SVM	58.70/99.38	57.97/94.67	54.35/92.00	44.93/43.67
Linear-SVM	39.13/36.00	55.07/49.33	39.13/36.00	39.13/36.00
RF	59.42/100.00	64.49/100.00	59.42/89.67	42.75/73.33
KNN	41.30/100.00	57.25/97.00	55.07/92.00	32.61/62.00

Strategie	Classifer			
	A1 ∪ A2	A1 ∪ A3	A2 ∪ A3	A1 ∪ A2 ∪ A3
Sigmoid-SVM	57.25/79.67	58.70/78.67	60.87/83.33	57.25/84.00
Rbf-SVM	60.87/92.67	55.80/89.67	61.59/94.33	63.04/93.67
Poly-SVM	60.87/94.67	57.25/93.00	64.49/95.00	63.04/96.00
Linear-SVM	39.13/36.00	39.13/36.00	39.13/37.00	41.30/38.33
RF	61.59/95.00	59.42/92.67	39.13/36.00	61.59/95.00
KNN	50.00/97.33	53.62/95.00	57.25/96.67	50.00/96.67

A1, A2, A3, and A4 are the feature vectors after maximum pooling with the pre-trained neu-ral network models ResNet50, DenseNet201, VGG16 and InceptionRenseNetV2, respectively

Meaning: Test acc/Validation acc (unit: %)

**Fig. 10:**
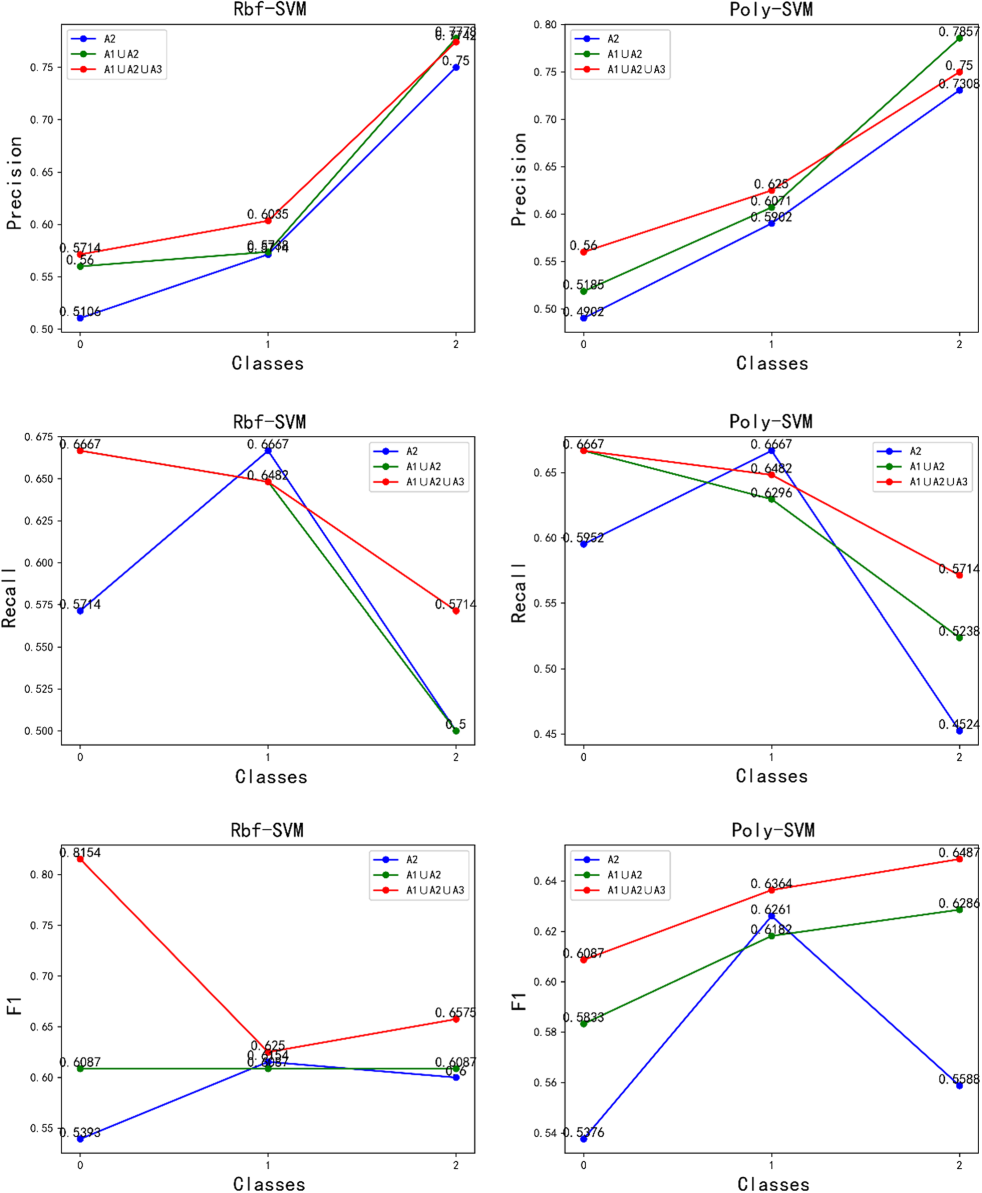
Classifier performance with three strategies**.** Note: A1, A2, A3 are the feature vectors after using full convolutional basis of the pre-trained neural network models ResNet50, DenseNet201 and VGG16, respectively as the feature extractor (∪ Denotes the serial fusion feature)

**Fig. 11:**
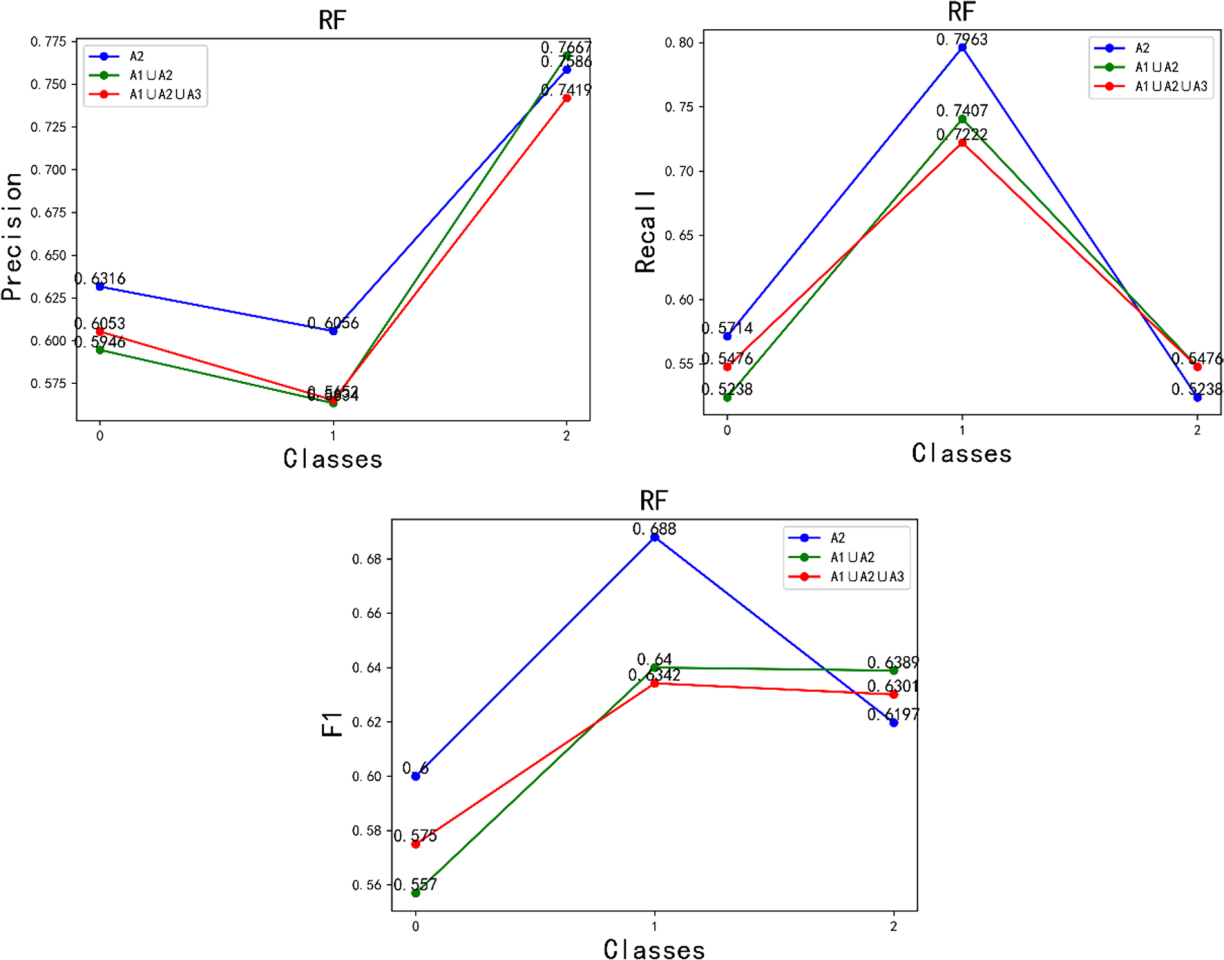
RF Classifier performance with three strategies. *Note* A1, A2, A3 are the feature vectors after using full convolutional basis of the pre-trained neural network models ResNet50, DenseNet201 and VGG16, respectively as the feature extractor (∪ Denotes the serial fusion feature)

In addition, we selected three types of feature vectors that performed well in the CNN feature analysis with the full convolutional basis, which were A2, A1 ∪ A2 and A1 ∪ A2 ∪ A3. Then we applied them with the top-performing Rbf-SVM, Poly-SVM and RF classifiers, respectively. We compared the accuracy, recall and F1 values with multiple strategies in the test set, and the comparison results are shown in Fig. 10. From Fig. 10 and Fig. 11, it can be seen that the fused features of the A1 ∪ A2 ∪ A3 strategy have the best complementarity, and the model has better generalization performance. It shows the best results in terms of precision rate, recall rate, and F1 value in the test set, thus indicating that the strategy of fusing the features of the pre-trained neural network model adopted in this study is reasonable. It is demonstrated that the features extracted by the pre-trained neural network VGG16, ResNet50 and DenseNet201 models are more complementary with the full convolutional basis, and the fused features improve the feature representation ability. Thus it can better extract the more complex image features among different liver tumor differentiation types.

In addition, we also explored the classification results of the fused features with the top-performing A1 ∪ A2 ∪ A3 strategy with the integrated learning classifier, and the experimental results are shown in Table 11, from which can be seen that the Stacking integrated learning model has the best classification results.

**Table 11:** Classifier performance with A1 ∪ A2 ∪ A3 fusion strategy

Classifier	Strategies	
	A1 ∪ A2 ∪ A3	
Stacking	Test acc/validation acc (unit: %)	63.04/94.67
	Images of precision, recall and F1 values	
Bagging	Test acc/validation acc (unit: %)	51.45/78.33
	Images of precision, recall and F1 values	
Gradient-boosting	Test acc/validation acc (unit: %)	57.97/88.33
	Images of precision, recall and F1 values	

### FuNet and performance analysis with different fusion strategies

**Fig. 12:**
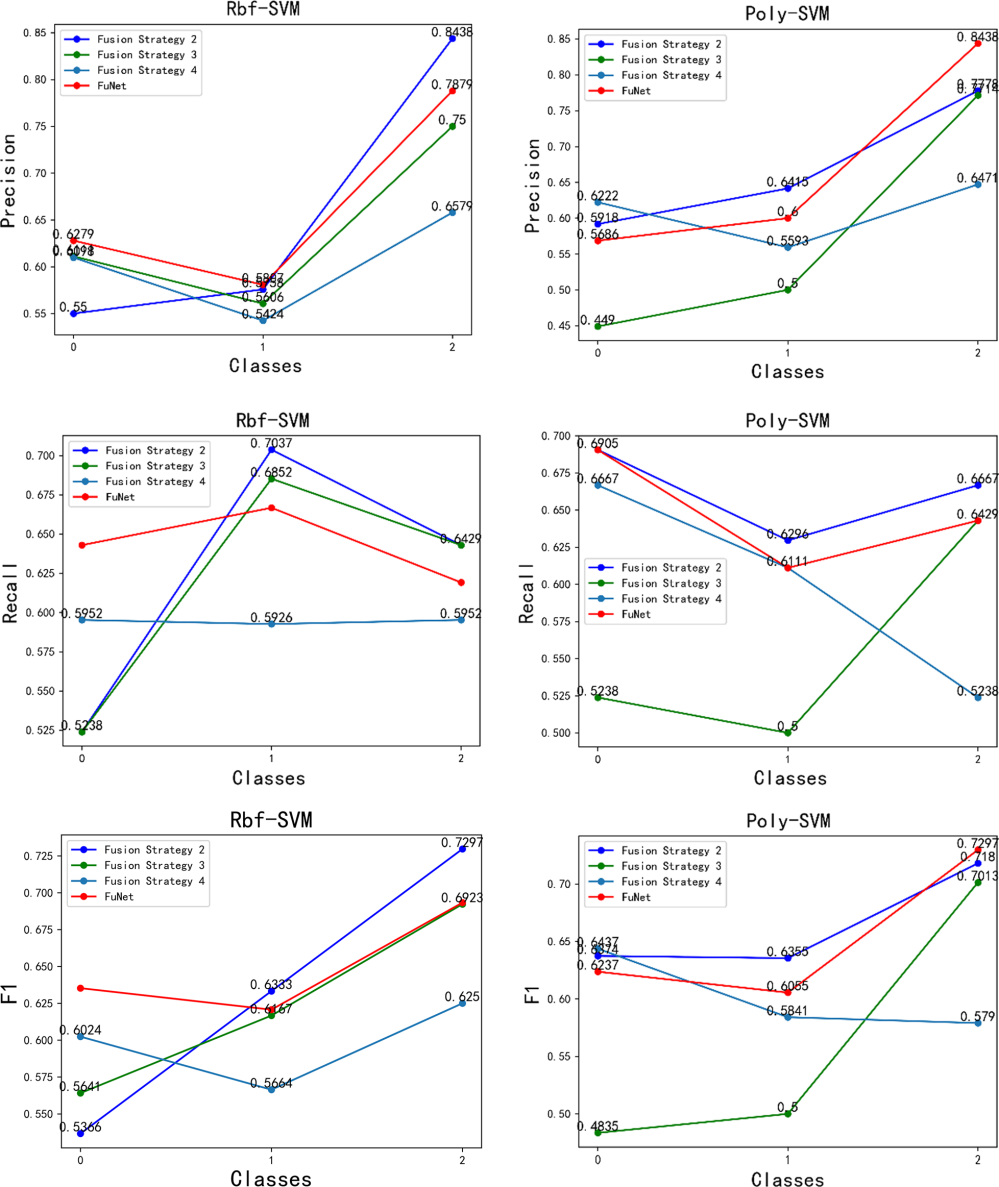
Classifier performance with four fusion strategies. Please refer to Table 8 and Fig. 7 for the specific name correspondence

**Fig. 13:**
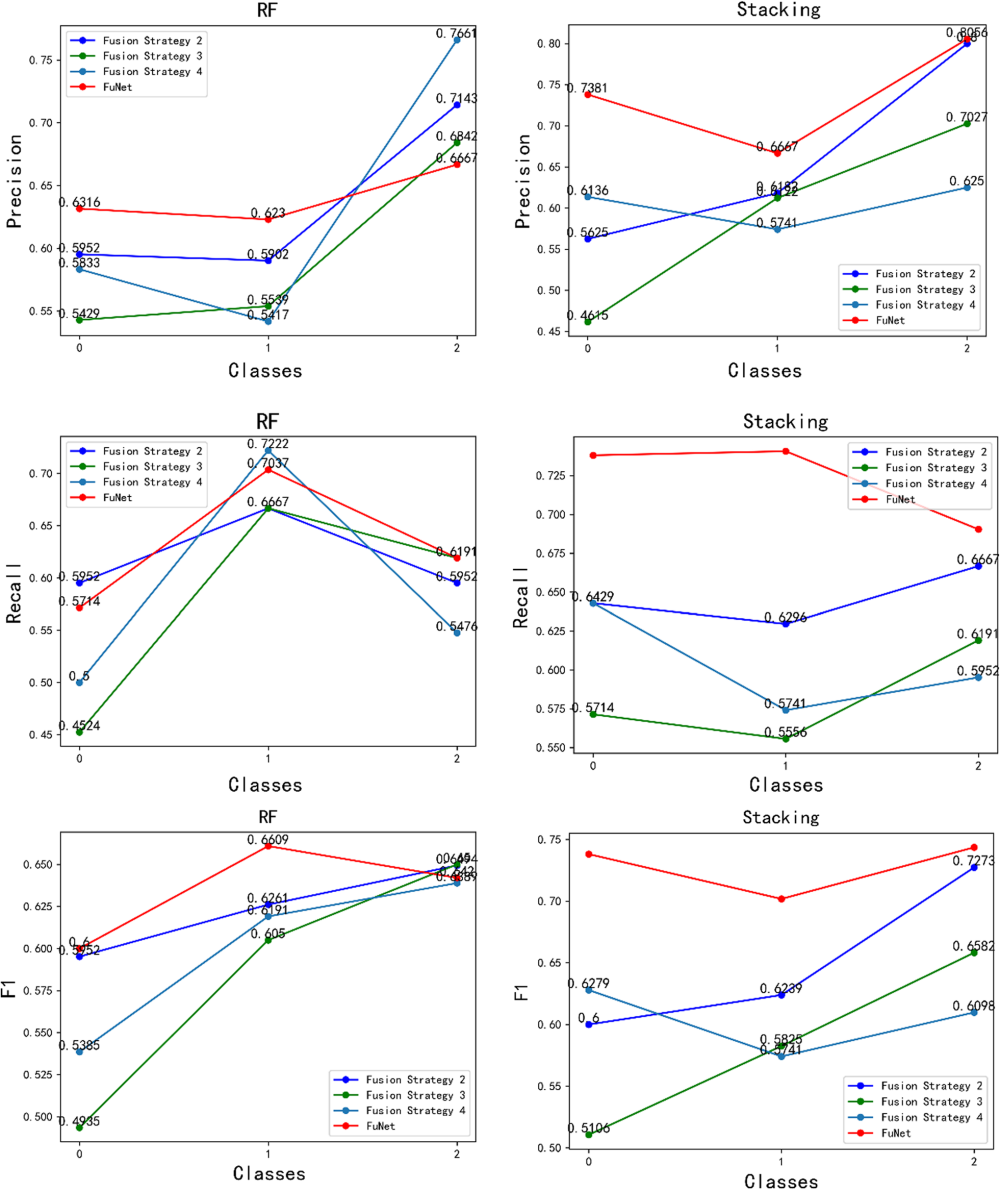
Classifier performance with four fusion strategies. Please refer to Table 8 and Fig. 7 for the specific name correspondence

**Fig. 14:**
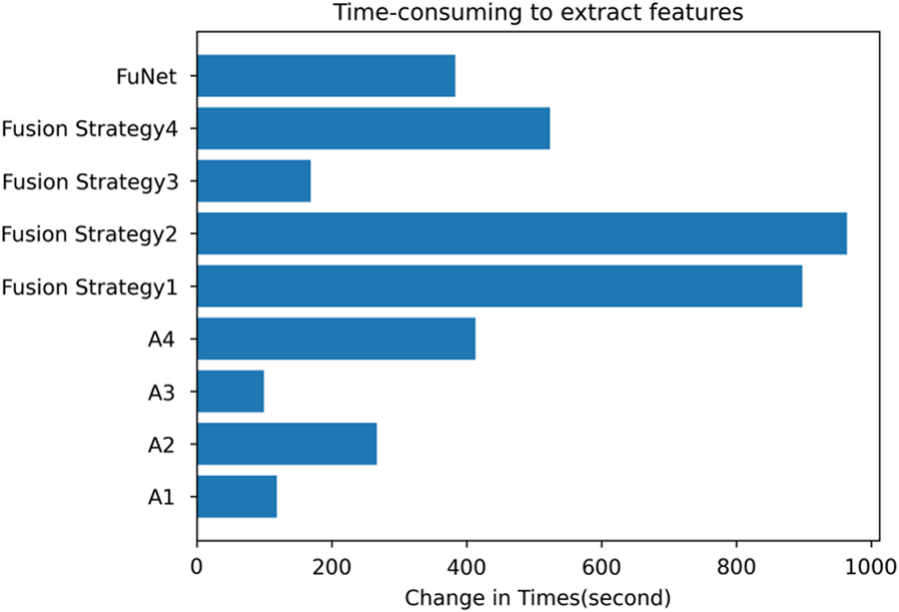
Time consuming of features extracting

We used different pre-trained models with different layers for feature extracting and fusing different fusion strategies as shown in Table 12. The FuNet fusion model, which combines the channel attention mechanism and the spatial attention mechanism, shows the best classification results with the Stacking integrated learning classifier in terms of validation set accuracy, test set accuracy, and time factor. It gets 72.46% accuracy in the test set and 94.33% accuracy in the validation set. In addition, to show the classification performance with FuNet fusion model, we also compared the accuracy, recall, and F1 value with different fusion strategies of the four models with excellent performance, Rbf-SVM, Poly-SVM, RF, and Stacking, and the length of time spent with different feature extraction and fusion strategies, and the experimental results are shown in Figs. 12, 13, and 14, respectively. We can see that with the stacking integrated learning model, the FuNet model obtains higher values in poorly differentiated, moderate differentiated, and well differentiated, which has better classification performance. The experimental results show that the FuNet model with fused channel attention and spatial attention extracts more complete and representative features of liver cancer tissue images, making the final Stacking classification model better to capture the subtle gaps in histopathological images of different differentiated types of liver cancer and achieve satisfactory results in classification accuracy.

**Table 12:** Classification accuracy of the fusion model (full convolutional basis)

Strategie	Classifer				
	Fusion strategy 1	Fusion strategy 2	Fusion strategy 3	Fusion strategy 4	FuNet
Sigmoid-SVM	59.42/77.67	65.22/74.33	63.77/62.66	57.97/68.33	66.67/81.67
Rbf-SVM	60.87/94.00	63.04/92.67	62.32/78.67	59.42/91.33	64.49/92.67
Poly-SVM	62.32/96.00	65.94/95.67	55.07/89.00	60.15/97.67	64.49/96.33
Linear-SVM	41.30/38.33	39.13/36.00	39.13/36.00	39.13/36.00	39.13/36.00
RF	60.87/94.00	62.32/94.33	58.70/94.00	60.15/94.67	63.77/95.67
KNN	50.00/96.67	46.38/91.67	42.75/93.33	42.75/91.63	42.75/93.00
Stacking	–	64.49/93.67	57.97/92.67	60.15/95.00	**72.46/94.33**
Bagging	–	73.19/72.67	43.48/44.67	56.52/76.00	71.01/80.00
Gradient boosting	–	65.22/88.00	52.17/82.00	64.49/87.00	60.87/91.00

Meaning:Test acc/validation acc (unit: %)

### ROC curve analysis

In this experiment, the scikit-learn module was used to calculate the ROC curves and AUC values, and two evaluation models, macro-average and micro-average, were specifically used. Macro-average focuses more on the performance of the classification model on the whole dataset but lacks in analyzing the performance of a specific category. Therefore, to reflect the model performance comprehensively, we also incorporate micro-averaging as a valid metric.

**Fig. 15:**
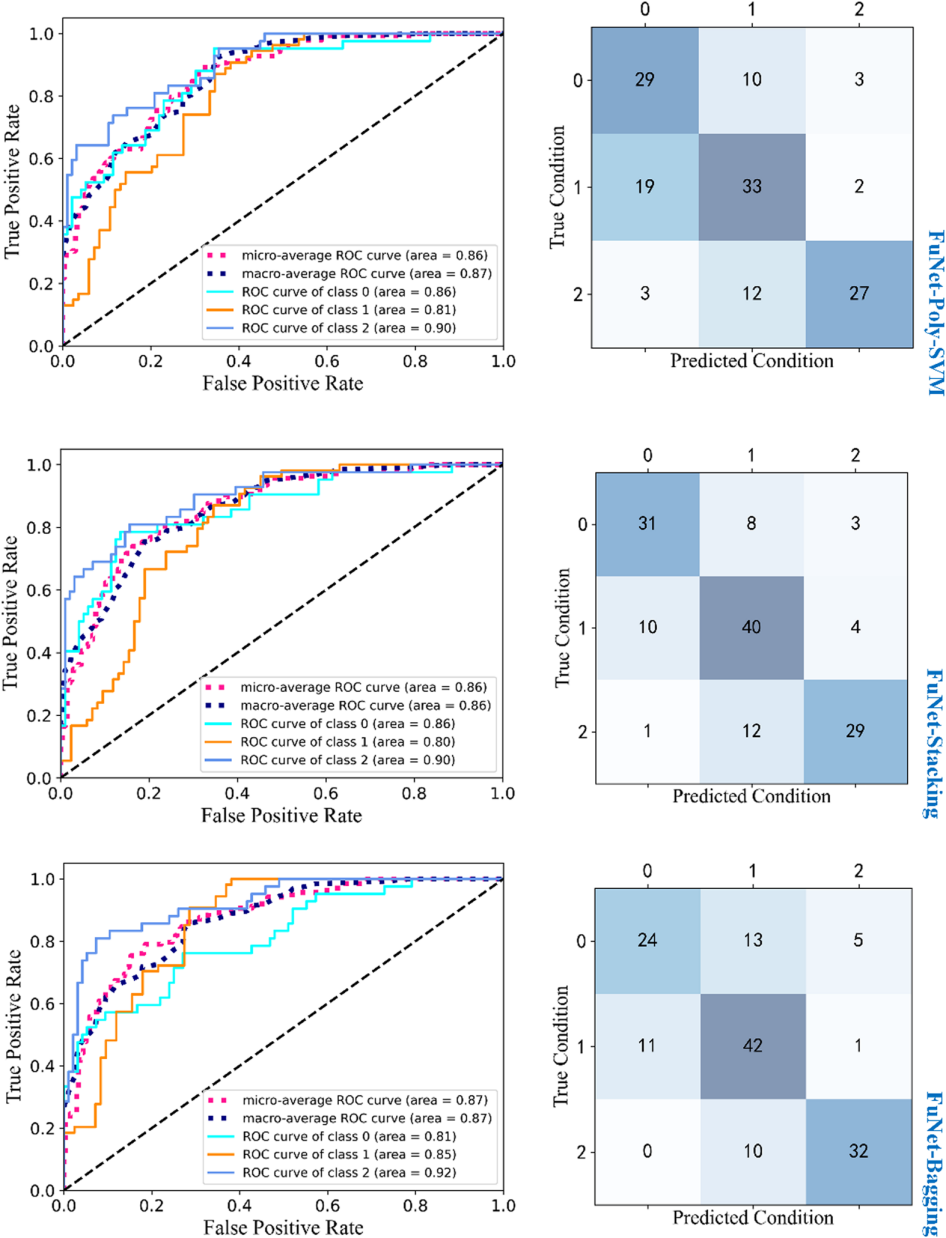
ROC curve and confusion matrix. Note: 0 means poorly differentiated, 1means moderate differentiated and 2 means well differentiated

We select the classification models with excellent classification performance with the fusion strategy: the ROC curves and confusion matrix of Poly-SVM, Stacking and Bagging classifiers with the FuNet model are shown in Fig. 15. The gap in the ROC curves of each model is not particularly large, and just minor differences. But it is gratifying to see that the AUC values of the FuNet model for the Poly-SVM, Stacking and Bagging classifiers are all above 0.8. In addition, by observing the confusion matrix, it can be seen that in the recognition results of poorly differentiated and highly differentiated categories, the recognition performance of the Stacking classifier is better than that of the Poly-SVM classifier. For the Bagging classifier with moderate differentiation of the category, the recognition result is better and the recognition accuracy is higher. In summary, through the comprehensive evaluation of both ROC curve and confusion matrix, the features extracted by the FuNet model have obtained the best classification results in the Stacking classifier, which is our best choice.

## Conclusion

In summary, we incorporated histopathological images of multi-differentiated subtypes of liver cancer and proposed a classification method for differentiation types of liver cancer based on Stacking classifier with deep feature integration of FuNet deep convolutional neural network. In our proposed framework, we used four pre-trained deep convolutional neural networks, ResNet50, VGG16, DenseNet201, and InceptionResNetV2, to extract deep features at different levels from histopathological images of multi-differentiated types of liver cancer. The extracted deep features are compared horizontally by multiple ML classifiers. Then the combinations of depth features that perform well on multiple ML classifiers are selected to stitch into a depth feature set and output the final classification results. In our experiments, we performed an adequate classification evaluation of liver cancer differentiation types using four pre-trained deep convolutional neural networks and nine different ML classifiers on a dataset of liver cancer histopathology images with multiple differentiation types. And the test set accuracy, validation set accuracy, running time with different strategies, accuracy, recall, F1 value, ROC curve and confusion matrix were used for adequate comparative evaluation. Our experimental results show that in the comparison experiments with different pre-trained neural network models using full convolutional bases, (1) the features extracted by VGG16, ResNet50 and Densenet201 models with full convolutional bases are more complementary, and the fused features of the three models improve the feature representation capability. As for running time, the fused A1UA2UA3 is a better choice when compared to the features fusing of InceptionResNetV2. (2) FuNet fusion strategy is a good choice, which covers both channel attention and spatial attention, and suppresses channel interference with less information. Meanwhile, it can clarify the importance of each spatial location by learning the weights of different locations in space, then apply it to the study of classification of multi-differentiated types of liver cancer. In addition, in most cases, the Stacking-based integrated learning classifier outperforms other ML classifiers in the classification task of multi-differentiation types of liver cancer with the FuNet fusion strategy after dimensionality reduction of the fused features by PCA features. In summary, our proposed new FuNet feature integration method helps to overcome the limitations of individual CNN models and outperforms feature fusion approaches at different levels with superior robust performance. These results suggest that our proposed method based on FuNet deep feature fusion and Stacking classifier is suitable for the classification of multi-differentiated types of liver cancer. Although the performance of our proposed FuNet model is promising, we still need to do further research to reduce the size and thus improve the classification performance of the model. To better target the automatic classification of liver cancer histopathological images, we will continue to collect liver cancer tissue samples from different institutions in our subsequent studies to further enhance our relevance in medical image classification tasks.

## Data Availability

The datasets generated and analyzed during the current study are not publicly available due to data privacy laws, but are available from the corresponding author on reasonable request.
